# Recent Progress on the Salt Tolerance Mechanisms and Application of Tamarisk

**DOI:** 10.3390/ijms23063325

**Published:** 2022-03-19

**Authors:** Qixin Duan, Zhihui Zhu, Baoshan Wang, Min Chen

**Affiliations:** 1 Shandong Provincial Key Laboratory of Plant Stress Research, College of Life Science, Shandong Normal University, Jinan 250014, China; qixinduan@yahoo.com (Q.D.); m15165085813@163.com (Z.Z.); 2 Dongying Institute, Shandong Normal University, No. 2 Kangyang Road, Dongying 257000, China

**Keywords:** application value, halophyte, salinization, salt tolerant, tamarisk

## Abstract

Salinized soil is a major environmental stress affecting plant growth and development. Excessive salt in the soil inhibits the growth of most plants and even threatens their survival. Halophytes are plants that can grow and develop normally on saline-alkali soil due to salt tolerance mechanisms that emerged during evolution. For this reason, halophytes are used as pioneer plants for improving and utilizing saline land. Tamarisk, a family of woody halophytes, is highly salt tolerant and has high economic value. Understanding the mechanisms of salt tolerance in tamarisk and identifying the key genes involved are important for improving saline land and increasing the salt tolerance of crops. Here, we review recent advances in our understanding of the salt tolerance mechanisms of tamarisk and the economic and medicinal value of this halophyte.

## 1. Introduction

Soil salinization is a global issue [1], with about 20% of the world’s available cultivated land estimated to be affected by salinity [2]. According to incomplete statistics from UNESCO (the United Nations Educational, Scientific and Cultural Organization, Paris, France) and FAO (the Food and Agriculture Organization of the United Nations, Rome, Italy), the total land area of the world is about 13.39 billion hectares, of which the saline-alkali land area is more than 800 million hectares [3], or more than 5.9% of the world’s total land area. In addition to the existing saline-alkali land, various factors such as climate change and excessive irrigation cause ongoing secondary salinization of arable land, resulting in more salinized land [4,5,6]. Based on previous statistics, approximately 70% of the land in northwest China is saline [7].

The problem of soil salinization has become one of the important issues discussed in the International Salinization Forum [8]. Soil salinization seriously affects global agricultural production [9,10]; the correlation between soil salinization and crop yield was studied and it was found that crop yields decreased linearly with increases in soil salt content [10,11]. It is speculated that by 2050, about 50% of the world’s arable land will become saline, which will greatly reduce agricultural production. Because of this, it is urgent to increase the area of arable land, improve agricultural production capacity, and accelerate the development and utilization of salinized land. Of all the measures available to reach these goals, increasing the salt tolerance of plants is the most economical and effective. Transgenic or genetic approaches based on advances in biotechnology and gene engineering can be used to improve the salt tolerance of crops. However, most of these improved crops can only survive on mildly saline soil (salt content below 100 mM) and might sustain low yields or die on most moderately and severely saline-alkali land [12]. So, the development and utilization of moderately and severely saline-alkali land is a huge challenge facing mankind.

Halophytes are highly salt-tolerant plants, defined by their ability to complete their life cycles at soil NaCl concentrations of no less than 200 mM for successive generations [13]. Studies have shown that halophytes are adapted to saline-alkali soil and that certain concentrations of NaCl can even promote their growth [14,15]. Therefore, saline-alkali soil is suitable for the cultivation of halophytes. However, halophytes account for only about 1% of all plants, and most have no economic or ornamental value, which restricts their development and utilization. Therefore, it is necessary to screen for and utilize valuable halophytes to develop moderately and severely saline-alkali land, which, because of its lack of cover vegetation, has a tendency toward desertification and ecological vulnerability [16,17,18]. Based on years of research, development, and application, most halophytes have been tested for this purpose [19]. Tamarisk (e.g., *Tamarix chinensis* Lour) is a many-branched shrub or tree that is a recretohalophyte, a halophyte that secretes salt. This typical woody halophyte can grow well on moderately and severely saline-alkali land, improving the environment and providing increased economic benefits [20,21,22]. In addition to being salt-stress-tolerant, tamarisk is also highly drought-tolerant, making it a good choice for the development and utilization of moderately and severely saline-alkali land. In this review, we focus on recent advances in understanding the salt tolerance mechanisms of tamarisk and the potential uses of this halophyte.

## 2. The Salt Tolerance Mechanisms of Tamarisk

**Figure 1:**
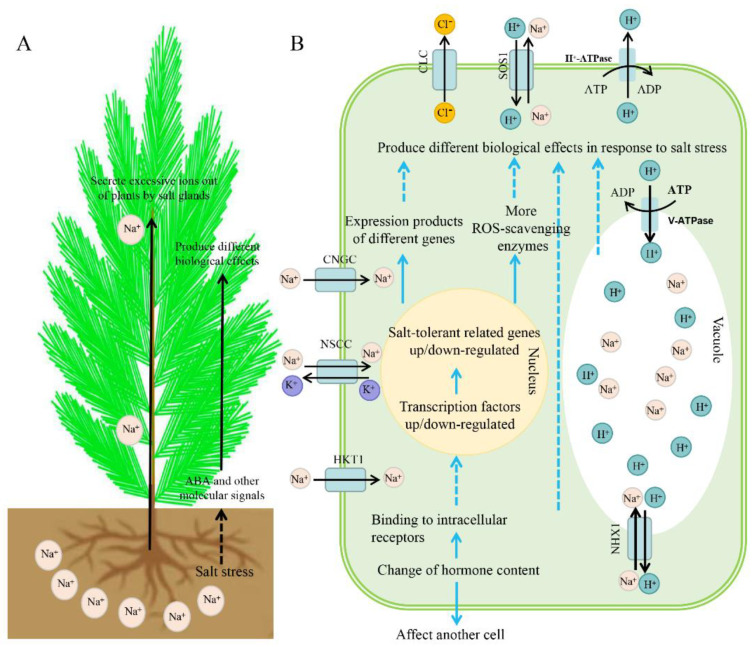
Strategies of tamarisk coping with salt stress. (**A**). The general strategies of tamarisk to deal with salt stress at the whole plant level. This involves the absorption of salt from roots and the removal of salt in the leaf by salt glands, as well as the transport of ABA and other signaling molecules from the roots to the aboveground under salt stress, (**B**). The general strategies of tamarisk in response to salt stress at the cellular level. Intracellular strategies for dealing with salt stress involve a complex regulatory network that helps cells reduce the damage caused by salt stress and remove salt from the cell to maintain normal physiological metabolism. In the figure, the solid line represents a direct effect, and the dotted line represents an indirect effect; CNGC, cyclic nucleotide-gated cation channel; NSCC, non-selective cation channel; CLC, chloride channel; HKT1, high-affinity potassium transporter; NHX1, tonoplast-located Na^+^/H^+^ antiporter; SOS1, salt overly sensitive 1 (plasma membrane-located Na^+^/H^+^ antiporter).

Tamarisk has two main options for adapting to a saline environment: salt avoidance and salt tolerance [23,24,25,26,27,28]. Tamarisk avoids salt damage by secreting salt via salt glands [23,24,28] and tolerates salt via many physiological and metabolic processes [25,26,27], such as osmotic regulation, scavenging of free radicals, cell detoxification, and protection of biological macromolecules (Figure 1). Maintaining metabolic homeostasis plays an important role in salt tolerance. The following sections review recent research on different aspects of the salt tolerance mechanisms of tamarisk.

### 2.1. Salt Ion Secretion

Recretohalophytes typically secrete salt ions that enter the plant through salt-secreting structures, mainly salt glands and salt bladders [29,30,31,32]. Dassanayake and Larkin (2017) [32] divided salt-secreting structures into four categories: salt bladders, unicellular salt glands, bicellular salt glands, and multicellular salt glands. Multicellular salt glands are the most complicated salt-secreting structures and generally consist of 4–40 cells, comprising collecting and secretory cells. Salt gland cells are enclosed in a cuticle-lined structure [33].

The salt glands of tamarisk are multicellular [23,24]. Early research focused on the structural and physiological characteristics of tamarisk salt glands. Thomson and Liu (1967) [23] showed that tamarisk salt glands have eight cells, of which two are collecting cells and six are secreting cells, and numerous plasmodesmata connecting the secretory cells with the collecting cells and the collecting cells with the adjacent mesophyll cells. Wei et al. (2020) [28] examined the salt glands of 11 species of Tamarix by differential interference microscopy and ultraviolet microscopy and found that they had only two secretory cells visible on the leaf surface. The cell walls in the salt gland cell fusion area were autofluorescent in tamarisk, similar to *Limonium bicolor* [33]. This autofluorescence, caused by the unique presence of ferulic acid in salt gland cell walls, can be used as a simple and reliable method to examine salt gland distribution and morphology [28,33,34]. Using this method [28], 11 Tamarix species were divided into three different types based on salt gland density, secretion rate per salt gland, and salt secretion capacity per leaf area. For all Tamarix species studied, salt secretion capacity and the size and density of salt glands significantly increased under NaCl treatment.

Salt secretion by salt glands plays a critical role in the salt tolerance of tamarisk. Several processes, including ion transport, movement through plasmodesmata, and vesicle transport, may be involved in salt secretion from salt glands [35,36,37]. Using specific inhibitors of cation pumps, channels, and transporters [38], it was shown that the salt glands in Tamarix species mainly secrete Na^+^ and K^+^. Ding et al. (2009) [30] showed that Ca^2+^ and H^+^-ATPase are involved in salt secretion from salt glands in *L. bicolor* under NaCl treatment. A large amount of transcriptome data suggested that ion transporters are involved in the salt secretion process [37,39].

There are many plasmodesmata between salt gland cells and between salt gland cells and adjacent mesophyll cells [23]. Plasmodesmata connect the cytoplasms of neighboring cells, constituting the symplastic transport pathway of plants. Molecular exchanges via the symplastic pathway are highly controlled [40]. Studies have shown that the intercellular channels traversing plasmodesmata provide a low-resistance pathway for many molecules, such as ions, RNAs, and hormones [41,42]. Thus, plasmodesmata can be an efficient symplastic transport pathway, especially for ions. Ma and Peterson (2001) [43] examined the ion transport pathway in roots from the epidermis to the stele. They analyzed the plasmodesmatal frequencies (which can be considered as representing the transport capacity through plasmodesmata) for all cellular interfaces ranging from the epidermis to the stelar parenchyma within the root and found that the radial transport of ions in the roots is really via plasmodesmatas. Zhang et al. (2022) [37] identified two plasmodesmata-localized proteins (BGs and PDCB5) involved in salt secretion from salt glands under salt stress.

During salt secretion by salt glands, many vesicles appear, suggesting that vesicular trafficking is involved in transporting and secreting salt. Lu et al. (2020) [36] downregulated the expression of the SNARE protein LbSYP61 (involved in vesicle transport) in *L. bicolor* using virus-induced gene silencing and showed that salt secretion by the salt glands decreased significantly. Through transcriptome and proteome analysis, Zhang et al. (2022) [37] identified a large number of vesicle-trafficking proteins involved in salt secretion by salt glands in *L. bicolor*. In tamarisk, based on salt gland distribution and ultrastructure, it was speculated that salt ions are transferred by vesicular transport from one excretory cell to the next until the ions are secreted from the plant [44]. However, the genes regulating the development and salt secretion ability of salt glands of tamarisk have not been well characterized. With continued progress in the molecular biology and biotechnology of tamarisk, we will be able to uncover the mechanism of salt secretion by salt glands in this plant.

### 2.2. Na^+^ Homeostasis and Osmotic Adjustment

In addition to secreting ions, another mechanism of salt tolerance in tamarisk is to maintain cellular ion homeostasis and osmotic balance [27]. Direct injury to plants by salt stress is caused by ion toxicity. Under salt stress, large amounts of Na^+^ enter plant cells, which can cause damage to macromolecules and membranes. Salt-tolerant plants can reduce the Na^+^ content of the cytoplasm by sequestering Na^+^ in the vacuole via Na^+^/H^+^ antiporters localized on the tonoplast and by secreting Na^+^ out of the cytoplasm via Na^+^/H^+^ antiporters (e.g., salt overly sensitive 1 [SOS1]) localized on the plasma membrane (PM). Two studies (Ma et al., 2019.; Che et al., 2019) [27,45] showed that TrSOS1 (from *Tamarix ramosissima*) had stronger Na^+^ efflux capacity than AtSOS1 (from *Arabidopsis thaliana*, a nonhalophyte). Overexpression of *TrSOS1* in cotton enhanced salt tolerance by maintaining a lower Na^+^ content and Na^+^/H^+^ ratio inside the plants. H^+^-ATPases localized on the PM (PM H^+^-ATPases) pump H^+^ out of the cell, creating a proton gradient across the plasma membrane that provides energy for SOS1 activity [45]. The same function is performed by vacuolar H^+^-ATPases (V-ATPases) to support Na^+^/H^+^ antiporters localized on the tonoplast [13]. The plant V-ATPase is a multisubunit complex and plays a role in salt tolerance in plants. Wang et al. (2020) [6] showed that homologous transformation of the *Th2CysPrx* gene (from *Tamarix hispida* Willd) into *T. hispida* improved salt stress resistance overexpression lines (Table 1).

Under salt stress, salt-tolerant plants generally accumulate organic compounds to osmotically adjust, protecting biomacromolecules and decreasing cytoplasmic water potential. For example, salt stress increases the proline contents in *T. chinensis* seedlings [55], and proline can be the main compound used for osmotic adjustments in tamarisk plants. Wang et al. (2017) [46] showed that transient overexpression of *ThNAC13* (a NAM, ATAF1/2, and CUC2 transcription factor gene from *T. hispida*) induced increased proline contents and improved salt tolerance. The nuclear protein ThNAC7 is another NAC transcription factor involved in salt tolerance in *T. hispida*. Transient overexpression of *ThNAC7* in *T. hispida* seedlings decreased reactive oxygen species (ROS) contents and increased proline contents, thereby increasing salt tolerance. Additionally, ThNAC7 can upregulate the expression of genes associated with stress tolerance and improve osmotic stress tolerance by increasing the content of osmotic regulatory substances and reducing ROS [47]. Zang et al. (2015) [48] suggested that ThZFP1 (a zinc finger protein from *T. hispida*) positively regulates proline accumulation in transgenic Arabidopsis and *T. hispida* plants. Furthermore, ThDof1.4 (a Dof protein from *T. hispida*) binds to a Dof motif in the *ThZFP1* promoter to activate *ThZFP1* expression. Further study showed that ThDof1.4 and ThZFP1 form a transcriptional regulatory cascade involved in increasing salt resistance in *T. hispida* by increasing proline levels and ROS-scavenging capability [49] (Table 1). Proline is not the only important osmotic adjusting substance. ThbZIP1 is a basic leucine zipper protein involved in abiotic stress responses in *T. hispida*. Transgenic tobacco plants overexpressing *ThbZIP1* increased their salt resistance by accumulating compatible osmolytes (soluble sugars and soluble proteins) and inducing the biosynthesis of soluble proteins.

### 2.3. Efficient ROS Scavenging Mechanism

Plants subjected to salt stress tend to have increased levels of ROS [56]. ROS have a dual effect on plants. At certain concentrations, ROS can act as signal molecules to improve the salt tolerance of plants and keep them alive; low concentrations of ROS are required for many important signaling reactions [57]. Baral (2019) [58] showed that ROS activated a downstream signaling pathway to increase the activities of ion transporters and reduce the concentration of Na^+^ ions in roots to help plants resist salt stress [58]. However, high concentrations of ROS can damage biological macromolecules, membranes, and basic biological processes, disrupting the normal metabolism and function of cells and even preventing the survival of plants [59,60]. ROS-scavenging enzymes in plants include SOD (superoxide dismutase), CAT (catalase), POD (peroxidase), APX (ascorbate peroxidase), GPX (glutathione peroxidase), and GST (glutathione S-transferase [48]. The activity or level of expression of these antioxidant enzymes increases in salt-stressed plants [56]. Furthermore, increased activity of antioxidant enzymes is often considered to increase plant tolerance to certain concentrations of salt [60,61].

In addition to salt secretion, an effective ROS-scavenging system is also key to the salt tolerance of tamarisk. Yang et al. (2014) [59] found that overexpressing *ThGSTZ1* (a *GST* gene from *T. hispida*) in Arabidopsis increased the survival rates of the transgenic plants under salinity stress. At the same time, these transgenic plants exhibited increased levels of GST, GPX, SOD, and POD activities, along with decreased malondialdehyde (MDA) contents and ROS levels under salt stress conditions. Transiently overexpressing *ThGSTZ1* in *T. hispida* significantly increased GST and GPX activities and improved ROS-scavenging ability under high-NaCl conditions [59]. As discussed earlier, Zang et al. (2015) [48] showed that the *T. hispida* zinc finger protein ThZFP1 is involved in salt stress resistance. Overexpression of *ThZFP1* in *T. hispida* plants using a transient transformation system induced the expression of POD and SOD genes, leading to enhanced SOD and POD activities and thus increased ROS-scavenging capability under salt stress. A 2-Cys peroxiredoxin gene from *T hispida* (*Th2CysPrx*) is also involved in salt stress tolerance [6] (Table 1). Further research showed that Th2CysPrx increased the salt tolerance of plants by increasing the activities of antioxidant enzymes and enhancing ROS removal. Moreover, Th2CysPrx upregulated the expression of *ThGSTZ1*, *ThGPX*, *ThSOD*, and *ThPOD*. ThTrx5 (a *T. hispida* thioredoxin) is also involved in increasing SOD, POD, CAT [61], and glutathione levels [62], as is ThSAP30BP (an SAP30-binding protein from *T. hispida*). [51]

The regulation of ROS content may be important for salt tolerance in tamarisk. Liu et al. (2021) [52] showed that ThSOS3 (*T. hispida* SOS3) plays an important role in ROS homeostasis in tamarisk. Transient overexpression of *ThSOS3* in *T. hispida* plants significantly increased their ROS-scavenging capability and antioxidant enzyme activities (Table 1). Wang et al. (2021) [53] constructed a salt-stress-regulated gene library for *T. hispida* and identified 1224 potential salt tolerance genes. Of these 1224 genes, 21 were randomly selected for further analysis, and 19 of these were involved in salt tolerance in *T. hispida*. Overexpression and knockdown of the 19 genes in *T. hispida* significantly decreased and increased, respectively, the ROS levels in the transformed plants compared to the control under salt stress. These results suggested that these genes are involved in scavenging ROS in *T. hispida* under salt stress.

NAC transcription factors are also involved in ROS scavenging in *T. hispida*. Wang et al. (2021) [53] showed that overexpressing *ThNAC12* (a *T. hispida* NAC transcription factor gene) in Tamarix and Arabidopsis enhanced their salt tolerance by increasing the ROS-scavenging capability and antioxidant enzyme activities under salt stress. Further research found that ThNAC12 increased salt tolerance in plants through direct regulation of ThPIP2;5 (aquaporins 2:5 which is a plasma membrane intrinsic protein from *T. hispida*) expression in *T. hispida*. *ThWRKY4* (a *T. hispida WRKY* gene) is highly induced by salt stress and can be regulated by ABF (ABRE binding factor) and Dof (DNA binding with one finger) transcription factors. ThWRKY4 increases the salt tolerance of *T. hispida* by increasing the activities of antioxidant enzymes and decreasing the levels of ROS [54]. Further study showed that the expression of *ThWRKY4* was activated by ThHSFA1 (a heat shock transcription factor from *T. hispida*) and that ThHSFA1 can bind to the heat shock element (HSE) of the *ThWRKY4* promoter. *ThHSFA1*-overexpressing *T. hispida* displayed similar salt tolerance phenotypes to those of *ThWRKY4*-overexpressing plants: enhanced salt tolerance, increased antioxidant enzyme activities, and reduced ROS levels under salt stress [63].

## 3. Application of Tamarisk

**Figure 2:**
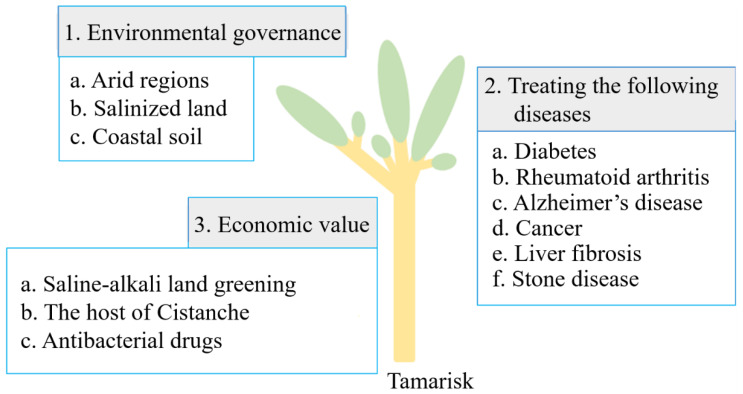
Main application value of tamarisk in three aspects.

Tamarisk has many applications due to its high salt tolerance and economic and medicinal value (Figure 2).

### 3.1. Significance of Tamarisk to the Environment

Because tamarisk is drought- and salt-tolerant, it can be used to build windbreaks to resist erosion by wind and sand in arid regions; it also can be planted on salinized land to improve the local ecological environment [64]. Many trees barely survive on severely saline land, so coastal saline-alkali areas are almost barren due to the lack of suitable trees. However, because of its high salt tolerance, tamarisk is the preferred tree species for greening of coastal saline land. Under these conditions, tamarisk can increase land cover, inhibit soil resalinization, decrease soil salinity, and improve soil quality and the local environment [65]. In addition, tamarisk can purify the environment by decreasing the levels of some pathogens and removing organic compounds where it is grown [66]. Tamarisk also can absorb heavy metal ions, thereby repairing soil polluted by heavy metals.

The development and utilization of tamarisk are mainly concentrated in three aspects. Each aspect is divided into several small research directions, which are marked with numbers in lowercase in English in the boxes connected to each aspect.

### 3.2. Medicinal Value of Tamarisk

Tamarisk has been used as a medicine for hundreds of years by traditional Chinese doctors. An ancient Chinese medical book, *Compendium of Materia Medica*, records the functions of tamarisk, including its use for treating a variety of diseases and detoxification. In recent years, many studies involving tamarisk extracts support the medical value of tamarisk. Tamarisk is rich in phenols and flavonoids, which are believed to possess medical value because of their antioxidant, anti-inflammatory, and anti-cancer activities [67]. Tamarisk preparations used to treat disease showed no toxicity in animal tests, so tamarisk can be considered as a safe medicinal plant [68,69]. The following sections review the main uses of tamarisk to treat disease.

#### 3.2.1. Treatment of Diabetes

The inhibition of α-amylase is an important strategy for treating diabetes [70], (Tundis et al., 2010; Cardullo et al., 2019) [71] due to its role in the digestion of carbohydrates and blood sugar levels. Experiments on rats suggest that tamarisk extracts can inhibit the activity of α-amylase and thus be used in diabetes-related research [72]. In addition, an aqueous extract of tamarisk alleviated complications of diabetes in rats [73]. These results suggest the potential of tamarisk as a treatment for diabetes.

#### 3.2.2. Treatment of Rheumatoid Arthritis

Several factors affect the onset of rheumatoid arthritis (RA), including heredity and unhealthy habits, and possibly the environment and microorganisms [74]. Tamarisk is used by traditional Chinese doctors for the clinical treatment of RA. Recent studies have shown that tamaractam, a type of lactam found in tamarisk, is an active component that may be used to treat RA, much as the tamarisk herb was used in the past as a traditional Chinese medicine [75]. In addition, ramosissimin, a flavonol extract from Tamarix, can induce apoptosis of rheumatoid arthritis fibroblast-like synoviocytes (RA-FLSs) [76], supporting the value of ramosissimin as a treatment for RA [77,78].

#### 3.2.3. Treatment of Alzheimer’s Disease

Despite decades of research on Alzheimer’s disease, which have led to some hypotheses and various possible pathogenic mechanisms, there are no effective treatment options or specific medicines for this disease [79]. Previous work suggested that vitamins E and C lower the incidence of Alzheimer’s disease by counteracting free radicals [80,81]. Similarly, tamarisk extract contains antioxidants that reduce the damage caused to the brain by oxygen free radicals and thereby may help in the treatment of Alzheimer’s disease [82,83].

#### 3.2.4. Treatment of Cancer

Tamarisk contains some substances that inhibit cell proliferation. These substances are thought to inhibit the proliferation of cancer cells by blocking the cell cycle [84]. For example, syringic acid extracted from tamarisk can trigger a variety of biological effects, including cell cycle arrest and apoptosis, to inhibit the proliferation of rectal cancer cells [85]. Likewise, methylferulate from tamarisk shows activities against rectal cancer cells similar to those of syringic acid. Methylferulate plays a role not only in the regulation of the cell cycle and apoptosis of rectal cancer cells, but also in making these cells more susceptible to related medicines [86]. Although these advances illustrate the potential of using tamarisk in cancer treatment, it is still a long way from clinical application.

#### 3.2.5. Liver Protection

Liver fibrosis is a serious chronic disease that can be induced by many factors, including chronic liver disease. Late-stage liver fibrosis often endangers the patient’s life [87,88], and liver transplantation is currently the best treatment. Research into the molecular mechanisms of liver fibrosis reversibility may pave the way to developing a better treatment that can reverse liver fibrosis [89]. Recent experiments have shown that polyphenols extracted from tamarisk may have anti-fibrosis effects in animals [90]. In addition, a methanolic extract of tamarisk may help inhibit the carcinogenesis of hepatocytes in a variety of ways [91]. In rats with induced liver damage, a water–alcohol extract of tamarisk returned some biochemical indicators of live damage to normal levels, suggesting that the extract had hepatoprotective activity [6,92]. Therefore, tamarisk also has great research value in the development of hepatoprotective medicine.

#### 3.2.6. Prevention of Lithiasis

Lithiasis, such as the development of urinary stone disease, is often associated with the accumulation and deposition of calcium oxalate in the body [93]. In such cases, inhibiting the formation of calcium oxalate might prevent lithiasis. In vitro experiments showed that an acidic component of tamarisk extracts can effectively inhibit the formation of calcium oxalate [94].

### 3.3. Economic Value of Tamarisk

**Figure 3:**
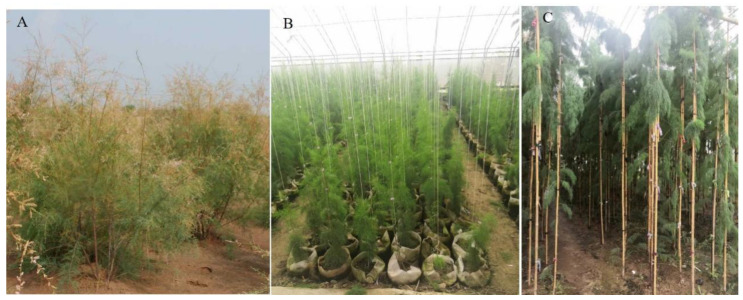
Arborization process of tamarisk seedlings in a greenhouse, (**A**). Three-year-old natural tamarisk seedlings, (**B**). One-year-old arborized tamarisk seedlings, (**C**). Three-year-old arborized tamarisk seedlings.

Tamarisk can be arborized through appropriate culture methods (Figure 3). This cultivation method was granted a patent in China in 2012 (patent number CN201210082425.2). Arborizing tamarisk overcomes its usual defects, such as soft branches, low degree of lignification, and no obvious trunk, producing a trunk that is straight and robust. This method is simple and feasible and can be used for the large-scale cultivation of tamarisk woodlands. Arborized tamarisk will be an important force for greening saline-alkali land.

For the rapid propagation of high-quality tamarisk, the method of clonal micropropagation should be applicated. This cultivation method was granted a patent in China in 2020 (patent number ZL201710566529.3).

Cistanche deserticola (Herba Cistanche, cistanche, Rou Cong-Rong), native to northwest China, is a parasitic plant that absorbs nutrients and water from its host. It has high medicinal value and is a sought-after plant for traditional Chinese medicine [95]. Tamarisk that is inoculated with cistanche can act as its host, making full use of tamarisk’s strong survival ability and supporting the growth of cistanche in all kinds of harsh environments. A patent (number CN201010134456.9) has been filed for this inoculation method, which makes it possible for cistanche to grow on saline-alkali land, providing a way to fully develop the economic value of Tamarix.

A variety of anti-inflammatory agents have been identified in an ethyl acetate extract of tamarisk, with potential for the development of anti-inflammatory drugs [96]. Polyphenols from tamarisk, hispidulin, cirsimaritin, and isorhamnetin, were suggested to have good inhibitory effects on some bacteria [97], which can inhibit their proliferation [98,99]. Tamarisk extracts showed inhibitory effects on bacteria in the mouth [100] and may provide a good way to develop safe oral antibacterial drugs.

Furthermore, tamarisk extracts can be used as a source of antioxidants and as a natural preservative in food production due to their antibacterial properties. Moreover, in industrial production, tamarisk biomass can be used as a resource for cellulase and amylase production [101].

## 4. Outlook and Prospects for Development

There are many species of tamarisk, with about 90 species worldwide and 18 species and 2 varieties in China [37]. In recent years, the development of new Tamarix varieties has made it difficult to accurately distinguish different Tamarix species only by their appearance and traits. Therefore, to accurately distinguish tamarisk species, a DNA fingerprint atlas of all Tamarix species should be established. Such an archive will provide technical support for intellectual property protection and orderly development of the market.

Tamarisk is a highly salt-tolerant plant, and exploring the key genes for salt tolerance in this plant will be helpful to elucidate its salt tolerance mechanism and improve the salt tolerance of crops. Although some salt tolerance genes have been identified in tamarisk [48,49,62,102,103], their functions have been verified only in heterologous systems, such as Arabidopsis, tobacco, and tomato, or by transient expression, neither of which can reveal the real functions and regulatory network of genes in tamarisk. Salt tolerance is a complex genetic and physiological trait, determined by more than just a few genes [104]. Whole-genome sequencing of tamarisk should be carried out to excavate key genes of salt tolerance, and an efficient genetic transformation system should be established to verify gene function and clarify the salt tolerance regulatory network in tamarisk. These findings can be applied to increase the salt tolerance of crops.

Although tamarisk has a strong ability to survive adversity, it should be noted that this ability can have both advantages and disadvantages. On the one hand, tamarisk can be used to improve the environment where conditions are not suitable for the growth of crops or other plants [50]. On the other hand, the strong ability of tamarisk to survive can make it invasive, potentially causing serious damage to local native ecosystems if appropriate care is not taken. Therefore, the use of tamarisk to improve the ecological environment requires a comprehensive consideration of a variety of factors, rather than random planting.

## Figures and Tables

**Table 1:** The genes from tamarisk were expressed in plants and enhanced salt tolerance of transformed plants by regulating Na^+^ homeostasis, osmotic adjustment, and ROS homeostasis.

Gene Resources	Transformed Plants	Contribution to Salt Tolerance	References
*TrSOS1* from *Tamarix ramosissima*	cotton	Na^+^ homeostasis	Che et al., 2019 [27]
*Th2CysPrx* from *Tamarix hispida*	*T. hispida*	Na^+^ homeostasis	Wang et al., 2020 [6]
*ThNAC13* from *T. hispida*	*T. hispida*	osmotic adjustment	Wang et al., 2017 [46]
*ThNAC7* from *T. hispida*	*T. hispida*	ROS homeostasis, osmotic adjustment	He et al., 2019 [47]
*ThZFP1* from *T. hispida*	*T. hispida*, *Arabidopsis*	ROS homeostasis, osmotic adjustment	Zang et al., 2015 [48]
*ThbZIP1* from *T. hispida*	tobacco	osmotic adjustment	Zang et al., 2017 [49]
*ThGSTZ1* from *T. hispida*	*T. hispida*, *Arabidopsis*	ROS homeostasis	Yang et al., 2014 [50]
*ThSOS3* from *T. hispida*	*T. hispida*	ROS homeostasis	Liu et al., 2021 [51]
*ThNAC12* from *T. hispida*	*T. hispida*, *Arabidopsis*	ROS homeostasis	Wang et al., 2021 [52]
*ThWRKY4* from *T. hispida*	*T. hispida*	ROS homeostasis	Zheng et al., 2013 [53]
*ThHSFA1* from *T. hispida*	*T. hispida*	ROS homeostasis	Sun et al., 2021 [54]
